# Dataset on perception of public college students on underage drinking in Nigeria

**DOI:** 10.1016/j.dib.2019.103930

**Published:** 2019-04-19

**Authors:** Olujide A. Adekeye, Emmanuel O. Amoo, Sussan O. Adeusi, Olufunke O. Chenube, Frederick Ahmadu, Joseph Idoko

**Affiliations:** a Covenant University, Department of Psychology, Ota, Nigeria; b Covenant University, Department of Demography & Social Statistics, Ota, Nigeria; c College of Education, Early Childhood Unit, Agbor, Nigeria; d Covenant University, Department of Sociology, Ota, Nigeria; e Covenant University Counselling Centre, Ota, Nigeria

**Keywords:** Alcohol, College, Underage drinking, Youths, Nigeria

## Abstract

Alcohol is the most widely used substance of abuse among youths in Nigeria. Underage drinking poses a serious public health problem in most colleges and despite the health and safety risk, consumption of alcohol is rising. Having recourse to the public health objective on alcohol by the World Health organization, which is to reduce the health burden caused by the harmful use of alcohol, thereby saving live and reducing injuries, this data article explored the nature of alcohol use among college students, binge drinking and the consequences of alcohol consumption. Secondary school students are in a transition developmentally and this comes with its debilitating effects such as risky alcohol use which affects their health and educational attainment [1], [2]. This data article consists of data obtained from 809 (ages 14–20 years) participants from selected schools in Ota, near Lagos State, Nigeria. For data collection, the youth questionnaire on underage drinking was employed. This data article presents information on participants' alcohol demographics. Analyses of the data can provide insights into heavy episodic drinking (HED), ever drinkers, prevalence of alcohol consumption, strategies to reducing alcohol use, reasons for underage drinking and effects of alcohol consumption. The data will be useful for public health interventions.

**Table:** Specifications Table

Subject area	Psychology
More specific subject area	Counselling Psychology, Health Psychology
Type of data	Tables
How data was acquired	Use of questionnaire for data collection
Data format	Raw and analyzed (descriptive statistics)
Experimental factors	Cross sectional research design using the youth questionnaire on underage drinking
Data source location	Surveys were conducted among college students in Ota, Nigeria
Data accessibility	Data is included in this article
Related research article	Adekeye OA, Adeusi SO, Chenube OO, Ahmadu FO, Sholarin MA. Assessment of Alcohol and Substance Use among Undergraduates in Selected Private Universities in Southwest Nigeria. IOSR Journal of Humanities and Social Science (IOSR-JHSS) 2015 20(3): 1–7. http://www.iosrjournals.org/iosr-jhss/pages/20%283%29Version-2.html.

**Table:** 

**Value of the Data** ✓ The data on the strategies to reducing underage alcohol consumption in Nigeria can be compared with those from other Africa nations and the global community ✓ The data can be useful in analyzing gender differences in the volume of alcohol consumed ✓ The data can also be useful in analyzing age difference in the volume of alcohol consumed ✓ The nature of the data may serve a heuristic basis for alcohol research ✓ The data can be used by counselling psychologist serving senior secondary school (High school) students ✓ The data can assist with planning for public health interventions

## 1 Data

**Fig. 1:**
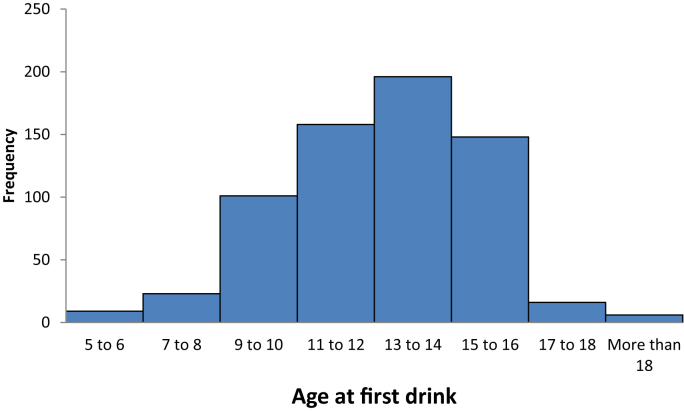
The histogram showing age at first drink.

Of the 809 students surveyed, 657 (81.2%) reported having drank alcohol. About half of the students (330 [50.2%]) had their first drink between ages 14 and 17 while 253 (39%) had their first drink between ages 10 and 13 years (see Fig. 1). Comparatively, in the United States, by age 15, 33% of teens have had at least 1 drink and about 60% of teens by age 18 have had at least 1 drink [3], [4]. 54 of the respondents reported to having more than five drinks in a single drinking episode, with 34 of these respondents saying they had done so within the last month. Data on the frequency of alcohol consumption show that 154 respondents drink at least once a week, 427 consume alcohol at least once a month while 76 respondents drink alcohol more than once a month (Table 1). Five cross tabulations were presented on gender and age responses to items on ever drank alcohol, frequency of drinks, problem of alcohol consumption, participant's perception on drinking and driving and whether alcohol consumption has increased, decreased or remained at the same level of consumption. The analyses are in Table 2a–e.

**Table 1:** Frequency of alcohol consumption.

Frequency of drink	At least once a week	At least once a month	More than once a month
Frequency	154	427	76

**Table 2:** a) Crosstabulation of ever drank alcohol by gender and age of respondents. b) Crosstabulation of alcohol consumption by gender and age of respondents. c) Crosstabulation of gender and age responses on problem of alcohol consumption. d) Crosstabulation of gender and age responses on problem of drinking and driving. e) Crosstabulation of gender and age responses on prevalence of alcohol consumption.

		Ever Drank		Total
		Yes	No	
Gender	Male	438	115	553
	Female	219	37	256
Total		657	152	809
Age Group	14years	73	31	104
	15–17years	497	71	568
	18–20years	87	50	137
Total		657	152	809

		Frequency of alcohol consumption n = 657			Total
		Once a Week	Once a Month	More than once a Month	
Gender	Male	99	287	52	438
	Female	55	140	24	219
Total		154	427	76	657
Age Group	14years	36	34	3	73
	15–17years	93	348	56	497
	18–20years	25	45	17	87
Total		154	427	76	657

		Alcohol consumption is … n = 754			Total
		Serious problem	Not a problem	Minor problem	
Gender	Male	447	34	29	510
	Female	192	20	32	244
Total		639	54	61	754
Age Group	14years	72	10	8	90
	15–17years	491	21	24	536
	18–20years	76	23	29	128
Total		639	54	61	754

		Drinking and driving n = 755			Total
		Serious problem	Not a problem	Minor problem	
Gender	Male	450	27	36	513
	Female	199	16	27	242
Total		649	43	63	755
Age Group	14years	72	7	12	91
	15–17years	485	24	31	540
	18–20years	92	12	20	124
Total		649	43	63	755

		Prevalence alcohol consumption is … n = 729			Total
		Increased	Decreased	Stayed the same	
Gender	Male	341	119	34	494
	Female	180	35	20	235
Total		521	154	54	729
Age Group	14years	53	23	14	90
	15–17years	402	101	23	526
	18–20years	66	30	17	113
Total		521	154	54	729

Respondents were given some potential strategies for reducing underage drinking and were asked to pick which of the strategies or approaches they would support in the quest to decreasing alcohol use by the underage. Table 3 provides a summary of their responses [1], [5]. We also asked respondents about what they thought some of the negative consequences of alcohol consumption were and the most common answers were “been driven by drunk driver”, “being absent from school”, and “been drunk at a party” (Table 4). Our data revealed that the majority of students obtained alcohol from bars or restaurants (Table 5) and in Table 6, the most common answer they gave for why they drank alcohol was “it enables them to enjoy a party” [3], [6], [7].

**Table 3:** Strategies to reducing alcohol consumption.

Approaches to decreasing alcohol use	Frequency/(%)	Rank
Alcohol educational interventions in schools	588 (72.7%)	1st
Use of mass media to advance Alcohol education	541 (66.9%)	2nd
Ban on alcohol advertising	490 (60.6%)	3rd
Improved law enforcement	402 (49.7%)	4th
Lectures by rehabilitated Alcohol users	384 (47.5%)	5th
More punishment	247 (30.5%)	6th
Suspending driving permit/license of drunk drivers	235 (29.0%)	7th
Alcohol-free recreational centres	200 (24.7%)	8th

**Table 4:** Negative Consequences of Alcohol consumption.

Negative Consequences	Frequency/(%)
Been driven by drunk driver	104 (24.4%)
Been absent from school	97 (22.7%)
Been drunk at party	86 (20.0%)
Been drunk at school	28 (6.6%)
Driving after drinking alcohol	27 (6.3%)
Had an injury	26 (6.0%)
Performing poorly in school	21 (5.0%)
Having family problems	21 (5.0%)
Been arrested	17 (4.0%)

**Table 5:** Places where Youths obtain Alcohol.

Where alcohol is obtained from…	Frequency/(%)
Bar/restaurant	322 (39.8%)
Liquor store	175 (21.6%)
Friends/relatives	169 (20.9%)
Parent's home	107 (13.2%)
Supermarket/convenience store	16 (2.0%)
Others	20 (2.5%)
Total	809 (100.0%)

**Table 6:** Reasons for youths alcohol consumption.

Youths drink because	Frequency/(%)
It enables them enjoy a party	525 (26.0%)
Peer influence and acceptance	485 (24.2%)
Relieves depression	478 (23.8%)
Boredom	318 (16.0%)
They want to stand up to authorities including parents	197 (10.0%)

## 2 Experimental design, materials and methods

**Fig. 2:**
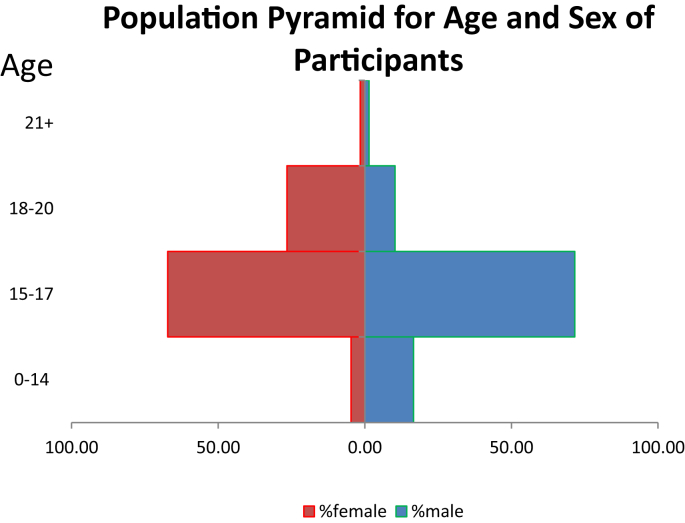
Population pyramid showing age of participants by gender.

We used a cross-sectional survey for this study on adolescent drinking in Nigeria. This dataset involved 809 students from some selected senior secondary schools in Ota, a sub-urban location in Southwest, Nigeria. Fig. 2 shows the age breakdown of participants by gender. This was represented by a population pyramid. Participants were selected from across all core subject areas such as sciences, arts and humanities and business classes through stratified and simple random sampling, to cater for variables such as gender, age, living location, subject area and ethnicity. Indeed, of the 809 students surveyed, 618 were Yoruba, 142 were Igbo, and 16 were Hausa (the remaining 33 students reported their ethnicity as “other”). The inclusion criteria included that the school principal/parents must sign a consent form or provide assent in writing; the participant (student) must be in senior secondary school class, and agree to participate freely. A participant must also be at least 14 years of age and not more than 20 years. Those who did not meet these criteria were excluded from the current study. The students were assured of the confidentiality of their responses. The questionnaire forms were filled in the classes with no interactions allowed among the participants and no access to the filled questionnaire forms by the school administrators. For data collection, an adapted questionnaire on youth alcohol consumption was employed. This questionnaire had items on use of alcohol and perception of youths to underage drinking and it elicited the desired information from the participants. The first part of the questionnaire dealt with respondents socio-demographic details. In order to ensure the psychometric requirements of the scale as advocated by Ref. [8], the reliability of the instrument was established using a test-retest reliability method. It was administered to 30 secondary school students and a second administration after a three-week interval with a Cronbach's Alpha of 0.83. The research trajectory was therefore considered adequate for data gathering purposes. All statistical analyses were performed using excel and IBM SPSS statistical software (v. 22).
