# Supplementary material for: Dataset on perception of public college students on underage drinking in Nigeria
Source: Data Brief. 2019 Apr 19;24:103930. doi: 10.1016/j.dib.2019.103930 (PMC6502769; doi:10.1016/j.dib.2019.103930)
Supplement: Multimedia component 1 [file mmc1.docx]

**Conflicts of Interest**

We wish to confirm that there are no known conflicts of interest associated with this publication and there has been no significant financial support for this work that could have influenced its outcome.

We confirm that the manuscript has been read and approved by all named authors and that there are no other persons who satisfied the criteria for authorship but are not listed. We further confirm that the order of authors listed in the manuscript has been approved by all of us.

We understand that the Corresponding Author is the sole contact for the Editorial process (including Editorial Manager and direct communications with the office). He/she is responsible for communicating with the other authors about progress, submissions of revisions and final approval of proofs.

We confirm that we have provided a current, correct email address which is accessible by the Corresponding Author and which has been configured to accept email from Elsevier

Signed by all authors as follows:

Olujide A. Adekeye {signed}

Emmanuel O. Amoo {signed}

Sussan O. Adeusi {signed}

Olufunke O. Chenube {signed}

Frederick O. Ahmadu {signed}

Joseph Idoko {signed}

**Olujide Adekeye**

17 February 2019.
