# Supplementary material for: Dataset on perception of public college students on underage drinking in Nigeria
Source: Data Brief. 2019 Apr 19;24:103930. doi: 10.1016/j.dib.2019.103930 (PMC6502769; doi:10.1016/j.dib.2019.103930)
Supplement: Multimedia component 3 [file mmc3.docx]

**YOUTH QUESTIONNAIRE ON UNDERAGE DRINKING**

**Please respond to these questions as honestly as you can. Utmost confidentiality is assured. Do not write your name or put your phone number on this paper. Thank you.**

**Background Information**

1. What was your age on your last birthday? Less than 14 ____, 15-17 ____, 18-20 _____, 21+______

2. What is your sex? Male ___________ Female ___________

3. What is your ethnicity? Yoruba _______ Igbo ________ Hausa ________ Others ______

4. Where do you live? Name of city/town ___________________ State _____________

**Use of Alcohol**

5. Have you ever had alcoholic beverages like beer, wine, or spirit? Yes ____ No___ (skip to question 18)

6. About how old were you the first time you drank alcohol, not counting sips you might have had as child from an older person’s drink? ___________ Years old

7. How often do you drink alcohol? At least once a week _______ At least once a month ___________ Less than once a month _____________

8. Do you ever have five or more drinks of alcohol at a time? Yes ___________ No ___________

9. If “Yes,” have you done this in the last month? Yes ___________ No ___________

10. “Have you ever.... ?” (Tick all that apply to you)

__________Been absent from school

__________Been drunk at school

__________Performing poorly in school

__________Having family problems

__________Been arrested

__________Driving after drinking alcohol

__________Been driven by drunk driver

__________Been drunk at party

__________Had an injury

11. Do your parents permit you to drink alcohol in your home? Never ____ On special occasions only____

Under parental supervision______ Anytime I want to______

12. Do you discuss alcohol use with your parent(s)? Yes ___________ No ___________

13. Do your parents know how much you drink? Yes ___________ No ___________

14. Have your parents ever seen you drunk? Yes ___________ No ___________

15. Do you know of parents or adults who permit non-family members under the age of 21 to consume alcohol in their homes? Yes ___________ No ___________

16. How many times in the last two months has someone offered to give you, buy for you, or sell you alcohol? None _________ Once __________ 2-3 times___________ 4 or more times ___________

17. Have you successfully used a fake age to obtain alcohol? Yes ___________ No __________

18. Have you ever purchased alcohol without being asked for your age? Yes ______ No _______

**Perception of Alcohol Use by Other People**

19. Most people my age who drink, do so because… (Tick all that apply)

______It enables them enjoy a party

______Relieves depression

______They want to stand up to authorities including parents

______Peer influence and acceptance

______Boredom

20. Do you think alcohol use by underage youth is a... Serious problem ____ Not at all a problem_______ Minor problem _______

21. Within the past year, do you think heavy use of alcohol among people your age has... Increased ______ Decreased _______ Stayed the same _______

22. Who is responsible for contributing to the problem of alcohol use by youth under age 21?

(Tick all that apply)

___________ Parents

___________ Public agencies

___________ Alcohol outlets, such as liquor stores, bars and restaurants

___________ Advertising

___________ Youth themselves

___________ Other (write in)

___________ Don’t know

23. Do you think drinking and driving among youth is a… Serious problem ____ Not at all a problem_______ Minor problem _______

24. Do you know someone with an alcohol problem? Yes ______ No _______

25. If the response to question 24 was “Yes,” what was their relationship to you? Relative ___________ Non-relative (e.g., friend or acquaintance) ___________

26. Where is the primary source where people under the age of 21 obtain alcohol? (Select only one)

Parent’s home ___________ Liquor store___________

Bar/restaurant ___________ Supermarket/convenience store___________

Friends/relatives ___________ Other___________

27. Which of the following approaches would you support to decrease alcohol use by youth under the legal drinking age of 21?

(Check all that apply)

_____More punishment

_____Improved law enforcement

_____Alcohol educational interventions in schools

_____Mass media to advance Alcohol education

_____Alcohol-free recreational centres

_____Lectures by rehabilitated Alcohol users

_____Suspend driving permit/license

_____Ban on alcohol advertising

**Thank you.**
